# The Lymphocyte-to-Monocyte Ratio (LMR) as a Novel Biomarker for Cervical Cancer Risk Stratification in Conization Patients

**DOI:** 10.3390/jcm14176057

**Published:** 2025-08-27

**Authors:** Verita Szabó, Szabolcs Várbíró, Noémi Kalas, Balázs Vida, Zsófia Tóth, Lotti Lőczi, Barbara Sebők, Petra Merkely, Balázs Lintner, Nándor Ács, Attila Keszthelyi, Márton Keszthelyi, Richárd Tóth

**Affiliations:** 1 Department of Obstetrics and Gynecology, Semmelweis University, 1082 Budapest, Hungary; szabo.verita@gmail.com (V.S.); kalasnoemi@gmail.com (N.K.); vidabalazs.2000@gmail.com (B.V.); toth.zsofia99@stud.semmelweis.hu (Z.T.); keszthelyi.lotti.lucia@semmelweis.hu (L.L.); merkely.petra@gmail.com (P.M.); lintner.balazs.zoltan@semmelweis.hu (B.L.); acs.nandor@semmelweis.hu (N.Á.); toth.richard@semmelweis.hu (R.T.); 2 Workgroup of Research Management, Doctoral School, Semmelweis University, 1085 Budapest, Hungary; varbiroszabolcs@gmail.com (S.V.); sebok.barbara23@gmail.com (B.S.); 3 Department of Obstetrics and Gynecology, University of Szeged, 6725 Szeged, Hungary; 4 Department of Urology, Semmelweis University, 1082 Budapest, Hungary; keszthelyi.attila@semmelweis.hu

**Keywords:** cervical cancer, HPV, lymphocyte-to-monocyte ratio (LMR), systemic inflammation, prognostic marker

## Abstract

**Background:** Cervical cancer remains a major global health burden, particularly in regions with limited early detection. The Loop Electrosurgical Excision Procedure (LEEP) is commonly used to diagnose and treat cervical intraepithelial neoplasia (CIN). The lymphocyte-to-monocyte ratio (LMR) is a potential biomarker for cancer risk stratification. It reflects immune function and tumor-related inflammation. Lower LMR values suggest reduced antitumor immunity and increased tumor-promoting inflammation, which are linked to cancer development and progression. This study examines relationships between preoperative LMR and histopathological outcomes after LEEP. **Methods:** This retrospective study included 374 patients undergoing the LEEP for cervical dysplasia. Preoperative LMR values were compared across four histopathological categories: negative, low-grade, high-grade lesions, and invasive carcinoma. The Kruskal–Wallis test assessed group differences, with Mann–Whitney U tests for pairwise comparisons. ROC curve analysis (*n* = 369) evaluated LMR’s diagnostic performance, and logistic regression evaluated its independent predictive value. **Results:** LMR significantly differed across cytological (*p* = 0.04) and histological groups (*p* = 0.036). Post hoc analysis revealed significantly lower LMR in invasive carcinoma versus low-grade lesions in cytology and for both low- and high-grade lesions in histology. ROC analysis yielded an AUC of 0.680. An LMR cutoff <4.49 showed 82.6% sensitivity and 50.0% specificity. Stricter cutoff (<3.89) increased specificity (66.8%) but decreased sensitivity (60.9%). Both had high negative predictive values (97.7% and 96.2%) but low positive predictive values (9.9% and 10.9%). **Conclusions:** LMR may serve as a complementary biomarker to predict higher-grade cervical lesions and help rule out invasive disease, aiding patient triage in resource-limited settings.

## 1. Introduction

Cervical cancer ranks among the most prevalent malignancies globally and predominantly affects women in middle age [1]. The occurrence of cervical cancer is rapidly increasing in developing countries due to undetected and untreated high-risk human papillomavirus (HPV) infection [2]. Contributing risk factors include early onset of sexual activity, multiple sexual partners, and inconsistent condom use. HPV, transmitted sexually, targets the epithelial tissues of the cervix, as well as the vagina, vulva, penis, skin, and oropharynx in both sexes [3,4]. The disease affecting the cervix typically progresses from a precancerous stage known as cervical intraepithelial neoplasia (CIN), which is classified into three grades: CIN I (mild dysplasia), CIN II (moderate dysplasia), and CIN III (severe dysplasia or carcinoma in situ) [5]. Despite advancements in screening technologies, CIN is frequently diagnosed at advanced stages, primarily due to insufficient early detection and inconsistent follow-up care [6]. Loop Electrosurgical Excision Procedure (LEEP) conization is a diagnostic and therapeutic technique employed in the management of cervical intraepithelial neoplasia [7]. This procedure involves the excision of abnormal cervical tissue [8]. 

In recent years, hematological markers such as the lymphocyte-to-monocyte ratio (LMR), platelet-to-lymphocyte ratio (PLR), and neutrophil-to-lymphocyte ratio (NLR) have gained recognition as prognostic indicators reflecting the link between systemic inflammation response and tumor progression. In particular, studies have demonstrated that systemic immune–inflammatory indices, including the systemic immune–inflammation index (SII), provide valuable prognostic information on cervical cancer and other malignancies, supporting the relevance of similar ratios such as LMR [9,10,11]. These ratios have shown clinical relevance across gynecologic malignancies, including cervical, endometrial, and ovarian cancers [12]. Elevated NLR, in particular, has been associated with p16 positivity and the presence of malignancy, underscoring its potential utility as a diagnostic and prognostic biomarker [7,13]. 

The lymphocyte-to-monocyte ratio is calculated by dividing the absolute lymphocyte count by the monocyte count in peripheral blood, making it an easily accessible marker derived from routine complete blood counts [14]. Clinically, LMR has emerged as a valuable tool for assessing cancer progression. Monocytes, upon infiltrating tumor tissues, differentiate into M1 or M2 macrophages—collectively termed tumor-associated macrophages (TAMs)—which play a pivotal role in tumor development by promoting invasion and metastasis through the secretion of growth factors, proteolytic enzymes, and motility-associated proteins [15,16]. Consequently, elevated peripheral monocyte counts are generally associated with unfavorable outcomes. In contrast, lymphocytes are central to the host’s antitumor immune response, particularly through the activation of cytotoxic T cells that mediate direct tumor cell destruction [17,18]. Numerous meta-analyses involving over 20,000 patients with various solid and hematologic malignancies have demonstrated that a decreased LMR is significantly associated with poorer overall survival, highlighting its potential as a low-cost, non-invasive prognostic biomarker in oncology [19].

LMR may be a clinically reliable marker for predicting patient prognosis and clinical outcomes. Thus far, most studies investigating the prognostic value of LMR have focused on gastrointestinal malignancies [20,21,22,23], as well as breast [24,25] and laryngeal cancers [26,27]. The prognostic and diagnostic significance of the lymphocyte-to-monocyte ratio (LMR) has not been comprehensively explored in the context of gynecologic oncology. This study explores the relationship between LMR and disease progression in patients undergoing LEEP conization for cervical intraepithelial neoplasia. The aim of this study is to determine an optimal cutoff value for the lymphocyte-to-monocyte ratio (LMR) for predicting the presence of malignancy.

## 2. Materials and Methods

### 2.1. Patients

This retrospective observational study was conducted within the framework of the SCOPE study (Semmelweis University Conization and Inflammation Outcomes with Predictive Evaluation). The study was designed to explore the clinical, histopathological, and laboratory characteristics associated with outcomes following cervical conization. Medical records spanning three years (2021–2024) were meticulously reviewed, and a comprehensive dataset was compiled. This dataset integrated sociodemographic variables, detailed gynecological and obstetric history, clinical presentations, and laboratory results, aiming to identify potential prognostic indicators and enhance our understanding of the biological and clinical influencing factors and risk stratification.

**Figure 1:**
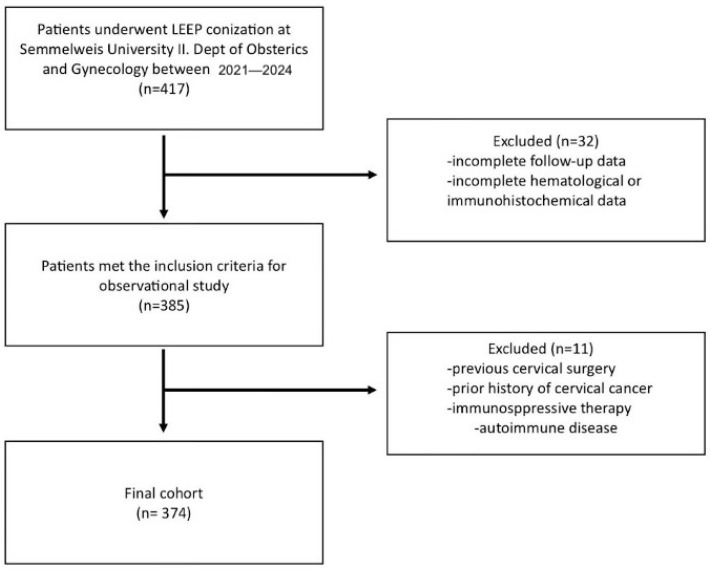
Patient selection flowchart. The figure outlines the sequential approach used to include and exclude participants from the study. Starting from the initial pool of individuals who underwent cervical conization, the process applies a series of clinical, cytological, histological, and data availability filters, ultimately defining the final study population.

A total of 417 patients who underwent conization at the 2nd Department of Obstetrics and Gynecology, Semmelweis University, were assessed for eligibility. Inclusion criteria mandated that participants had undergone conization specifically for the treatment of cervical intraepithelial neoplasia (CIN) and had complete clinical, histopathological, and laboratory data available. To minimize confounding effects on systemic inflammatory markers such as the lymphocyte-to-monocyte ratio (LMR), patients were excluded if they had a documented history of autoimmune disease, chronic inflammatory or hematological disorders, or acute infection at the time of blood sampling or were receiving immunosuppressive therapy. Additional exclusions included previous cervical surgery, prior diagnosis of cervical carcinoma, or the presence of other active malignancies. Patients with incomplete follow-up or missing hematological data were also excluded. After applying these rigorous selection criteria, 374 patients met the eligibility requirements and were included in the final analysis. The detailed patient selection workflow is depicted in Figure 1.

### 2.2. Characteristics

The sociodemographic data collected included the patient’s age at the time of surgery, calculated by subtracting the year of birth from the year the conization was performed, BMI, laboratory parameters, cytology, conization results, and HPV status. Laboratory parameters focused primarily on biomarkers associated with systemic inflammation, such as monocyte and lymphocyte counts. From these values, the lymphocyte-to-monocyte ratio (LMR) was derived. All laboratory analyses were conducted within one month before the surgical intervention to ensure clinical relevance and temporal proximity to the procedure.

The diagnostic screening for cervical dysplasia encompassed both cervical cytology results and HPV testing, with particular attention to the detection of high-risk HPV subtypes. Based on cytological screening findings, patients were stratified into four categories: Grade 1 (negative for intraepithelial lesion or malignancy); Grade 2 (low-grade squamous intraepithelial lesion [LSIL] and atypical squamous cells of undetermined significance [ASC-US]); Grade 3 (high-grade squamous intraepithelial lesion [HSIL], atypical squamous cells—cannot exclude HSIL [ASC-H], atypical glandular cells [AGC], and adenocarcinoma in situ [AIS]); and Grade 4 (invasive cervical cancer). Surgical data included the histopathological evaluation of conization specimens, which were graded using the same four-tier classification system. This harmonized grading approach was adopted to align cytological and histological categories with comparable clinical significance and management strategies. It also served to minimize diagnostic heterogeneity and mitigate the risk of statistical underpowering in smaller diagnostic subgroups. This methodology reflects recent recommendations in the literature that advocate for integrated, risk-based categorization due to overlapping morphological features in cervical cytopathology. It is intended for analytical consistency and does not replace standard clinical grading [15].

Ethical approval for this study was obtained from the Institutional Review Board of Semmelweis University (SE RKEB: 195/2024), following international ethical standards and institutional regulations governing patient confidentiality and data protection. The ethical approval was granted on 1 October 2024, and it is valid until 1 October 2026.

### 2.3. Data Management

For this retrospective analysis, patient data were systematically collected and entered into a dedicated electronic database developed specifically for the SCOPE study. The database included detailed records of sociodemographic characteristics, clinical parameters, and laboratory findings. Before statistical evaluation, data quality was thoroughly assessed through structured validation procedures. These included manual and automated checks for inconsistencies, as well as outlier detection using box plot visualizations to identify extreme values that could influence analytical outcomes. Missing data were addressed using predefined management protocols to ensure the completeness and reliability of the dataset, thereby maintaining its integrity for subsequent statistical analyses.

### 2.4. Statistical Analysis

Statistical analyses were performed using IBM SPSS Statistics for Windows, Version 25.0 (Released 2017; IBM Corp., Armonk, NY, USA). Violin plots with median lines, overlaid with jittered individual data points, were generated using R (version 4.3.2) with the ggplot2 package. Descriptive statistics, including mean, standard deviation, median, and range, were calculated for continuous variables. For categorical variables, frequencies and percentages were reported to describe the characteristics of the study population.

Comparisons of the lymphocyte-to-monocyte ratio (LMR) across different cytological and histopathological grades were conducted using the Kruskal–Wallis test, a non-parametric method suitable for comparing more than two independent groups. Post hoc pairwise comparisons were carried out using Dunn–Bonferroni correction to adjust for multiple comparisons. To complement the *p*-values and evaluate the practical significance of group differences, the effect size for each Kruskal–Wallis test was estimated using eta-squared (η^2^).

To investigate the relationship between LMR and histologically confirmed cervical malignancy, both univariable and multivariable binary logistic regression analyses were performed. The final multivariable model was fitted using the ‘Enter’ method in SPSS and included LMR and age as independent predictors. Candidate variables such as NLR, PLR, BMI, and HPV DNA positivity were initially considered based on prior evidence and clinical relevance; however, due to high rates of missing data and a lack of statistical significance, these were excluded from the final model to avoid overfitting and preserve statistical power. 

For all predictors retained in the final models, odds ratios (ORs) with 95% confidence intervals (CIs) were reported. Missing data were handled via listwise deletion (complete-case analysis), meaning that only cases with complete data across all variables were included in the multivariable analyses. The number of valid observations (N) included in each study is reported separately.

To further assess the discriminatory power of LMR, receiver operating characteristic (ROC) curve analysis was performed. The area under the curve (AUC) was calculated to evaluate model performance. Optimal cutoff values for LMR were identified using both the Youden index and the Closest Top Left method. Based on these thresholds, key diagnostic metrics—including sensitivity, specificity, positive predictive value (PPV), negative predictive value (NPV), positive and negative likelihood ratios (+LR and −LR), and overall diagnostic accuracy—were calculated.

Model fit was evaluated using Cox and Snell R^2^ and Nagelkerke R^2^ coefficients. The overall significance of the regression models was assessed using omnibus chi-square tests and ANOVA, where appropriate. As previously noted, missing data were managed through complete-case analysis, and the number of valid cases is indicated for each statistical test.

All statistical tests were two-tailed, and *p*-values < 0.05 were considered statistically significant. This comprehensive and methodologically rigorous statistical framework ensures the robustness and reliability of the findings. It also supports the clinical relevance of LMR as a potential non-invasive biomarker in the diagnosis and monitoring of cervical cancer.

## 3. Results

### 3.1. Patient Characteristics

Table 1 presents participant characteristics. The median age of the 374 patients was 40 years (interquartile range: 23–78 years). The median BMI was 22.85 (interquartile range: 14.64–46.48). The median of LMR was 4.35 (interquartile range: 0.32–18.52). Cytology and conization findings were classified into four grades: Grade I (negative), Grade II (LSIL, ASC-US), Grade III (HSIL, ASC-H, AGC, AIS), and Grade IV (cancer). The percentage distribution of the grades is shown in Table 1.

### 3.2. Relationship Between Laboratory Parameters and Cytology Results

Differences were assessed in the lymphocyte-to-monocyte ratio (LMR) across cytological severity grades using the Kruskal–Wallis test. The analysis revealed a statistically significant difference between the groups (χ^2^ = 8.307, df = 3, and *p* = 0.04), indicating that LMR values tended to vary by cytological abnormality. The distribution pattern of lymphocyte-to-monocyte ratio (LMR) values concerning the different categories of cytological grading is presented in Table 2.

No clear separation in LMR values was observed between the LSIL and HSIL categories. This may reflect the fact that these cytological classifications represent preinvasive stages, in which systemic inflammatory changes are less pronounced compared with invasive disease.

**Figure 2:**
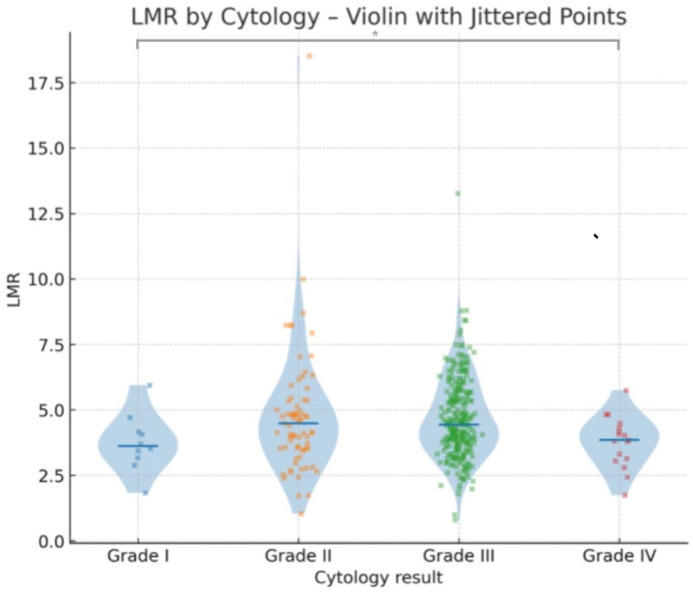
Violin plots showing the distribution of lymphocyte-to-monocyte ratio (LMR) values according to cytology results (Grades I–IV). Median values are indicated by horizontal lines, with jittered individual data points overlaid to visualize data spread and density. Asterisks (*) indicate statistically significant differences between groups (*p* < 0.05).

To further investigate pairwise group differences, Dunn–Bonferroni post hoc comparisons were performed (Table 3). A statistically significant difference was observed between Grade I and Grade IV (adjusted *p* = 0.01). No other comparisons remained significant after the Bonferroni correction (Figure 2).

**Figure 3:**
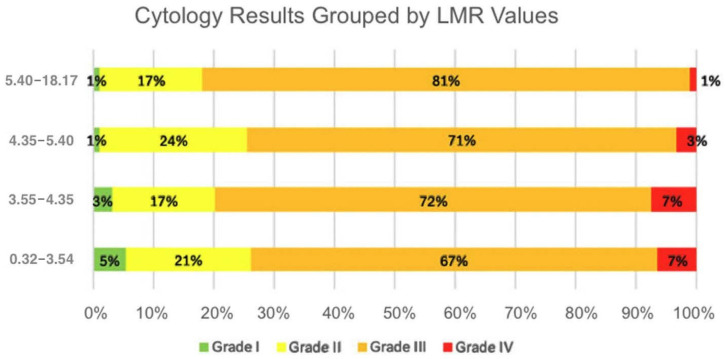
Distribution of cytological grades within lymphocyte-to-monocyte ratio (LMR) quartile groups. Each horizontal bar represents a specific LMR range, divided by the proportion of cases classified as Grade I–IV cytological abnormalities. The majority of higher LMR values were associated with lower cytological grades, whereas lower LMR values showed a higher proportion of Grade IV abnormalities.

A statistically significant downward trend in LMR values was observed across worsening cytological categories. Post hoc analysis further indicated that lower median LMR values are associated with more adverse cytological outcomes (Figure 3).

### 3.3. Relationship Between Laboratory Parameters and Conization (Histological) Results

A similar analysis was conducted using histological grades obtained from conization specimens. The Kruskal–Wallis test yielded a statistically significant difference in LMR values across the four histological categories (χ^2^ = 8.574, df = 3, *p* = 0.036).

**Figure 4:**
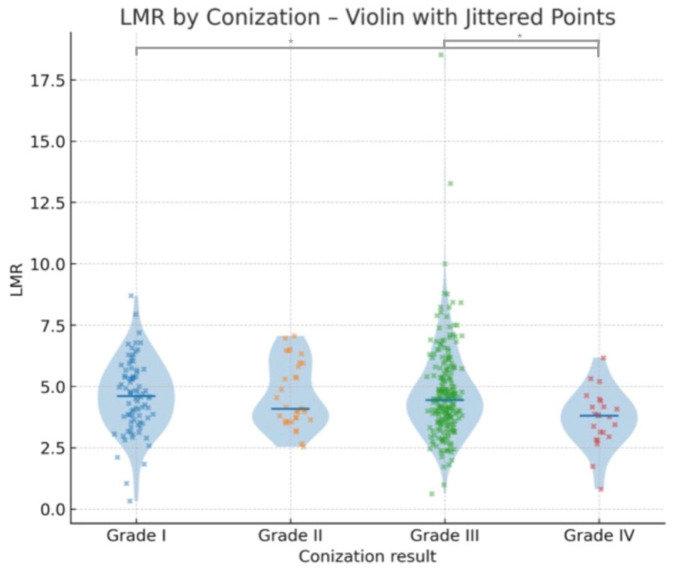
Violin plots showing the distribution of lymphocyte-to-monocyte ratio (LMR) values according to conization results (Grades I–IV). Median values are indicated by horizontal lines, with jittered individual data points overlaid to visualize data spread and density. Asterisks (*) indicate statistically significant differences between groups (*p* < 0.05).

**Figure 5:**
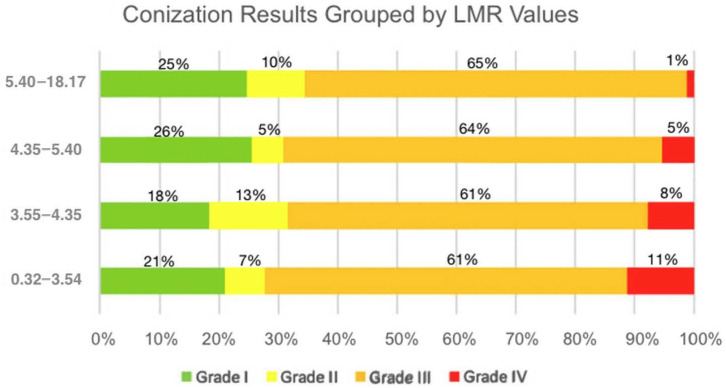
Distribution of conization results within lymphocyte-to-monocyte ratio (LMR) quartile groups. Each horizontal bar represents a specific LMR range, divided by the proportion of cases classified as Grade I–IV cytological abnormalities.

Dunn–Bonferroni-corrected post hoc comparisons revealed statistically significant differences between Grade I and Grade IV (adjusted *p* = 0.03) and between Grade III and Grade IV (adjusted *p* = 0.047) (Table 3). Other comparisons did not remain significant after correction. There is a significant decrease in LMR values in patients with invasive carcinoma (Grade IV) compared to lower histological grades (Figure 4). The results support the potential utility of LMR as a non-invasive marker in distinguishing high-grade histological lesions. The visual trend of decreasing LMR values with worsening histological categories aligns with the statistical significance, reinforcing the conclusion that low median LMR values were associated with poorer conization outcomes (Figure 5).

### 3.4. Relationship Between Laboratory Parameters and HPV DNA Positivity

**Figure 6:**
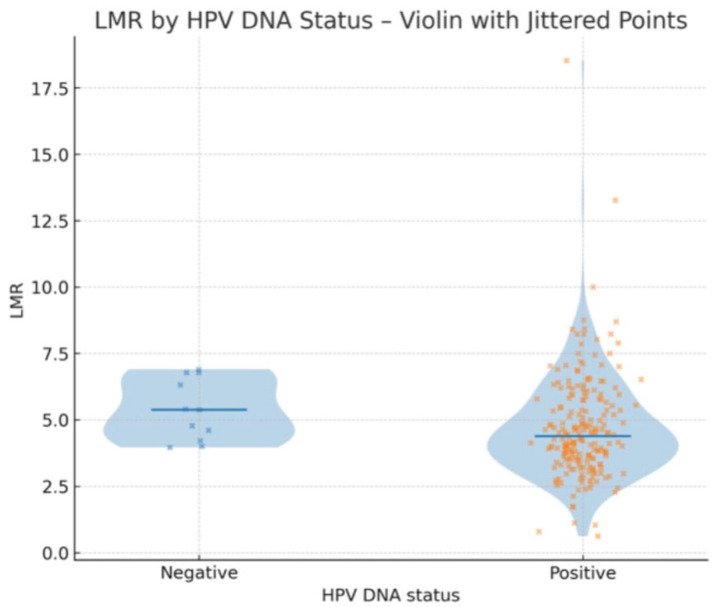
Violin plots showing the distribution of LMR values according to HPV DNA status.

Patients with HPV DNA positivity had a median LMR of 4.387, whereas those with HPV DNA negativity had a higher median LMR of 5.377. Although the difference did not reach statistical significance, a clear trend toward a lower LMR was observed in HPV-positive individuals. This suggests a potential link between HPV infection and systemic inflammatory response, warranting further investigation in larger cohorts (Figure 6).

### 3.5. Relationship Between Laboratory Parameters and Age

To identify independent predictors of histologically confirmed cervical cancer, a multivariable binary logistic regression analysis was conducted using the enter method. Candidate variables were selected based on biological relevance and the previous literature and included the lymphocyte-to-monocyte ratio (LMR), age, body mass index (BMI), and HPV DNA positivity.

Due to a high rate of missing data—particularly for HPV status—models including all predictors would have substantially reduced the effective sample size and increased the risk of overfitting. Moreover, preliminary analyses showed that only LMR and age were significantly associated with histological cancer diagnosis. 

Therefore, the final model was restricted to LMR and age to preserve statistical power and model stability. This model was statistically significant (χ^2^(2) = 17.043, *p* < 0.001), indicating that the predictors contributed meaningfully to the classification of cervical cancer. The model explained approximately 4.5% to 12.1% of the variance in cancer diagnosis (Cox and Snell R^2^ = 0.045; Nagelkerke R^2^ = 0.121).

Both LMR (B = −0.475, *p* = 0.007) and age (B = 0.058, *p* = 0.004) remained independent predictors. Higher LMR values were associated with a significantly lower likelihood of histologically confirmed malignancy (OR = 0.622; 95% CI: 0.444–0.871), while increasing age was associated with higher cancer risk (OR = 1.060; 95% CI: 1.019–1.103) (Table 4). Model calibration was assessed using the Hosmer–Lemeshow goodness-of-fit test applied to the final multivariable logistic regression model, which included LMR and age as predictors of histologically confirmed cervical malignancy. Predicted probabilities were grouped into deciles and compared with the observed frequencies of malignancy and non-malignancy. The χ^2^ statistic was 0.000 (*p* = 1.000), indicating perfect agreement between predicted and observed outcomes. These findings suggest that LMR, especially when combined with age, may serve as a simple and accessible biomarker for identifying patients at elevated risk for cervical cancer.

### 3.6. Diagnostic Performance

Receiver operating characteristic (ROC) analysis was conducted on data from 369 patients to assess the diagnostic performance of the lymphocyte-to-monocyte ratio (LMR) in detecting invasive cervical carcinoma. Outliers were assessed visually using violin plots with median lines, overlaid with jittered individual data points. Although a small number of statistical outliers were detected (LMR range: 0.32–18.52), none were deemed biologically implausible or likely to distort the results. Consequently, no data points were excluded from the ROC or regression analyses on the basis of extreme values.

**Figure 7:**
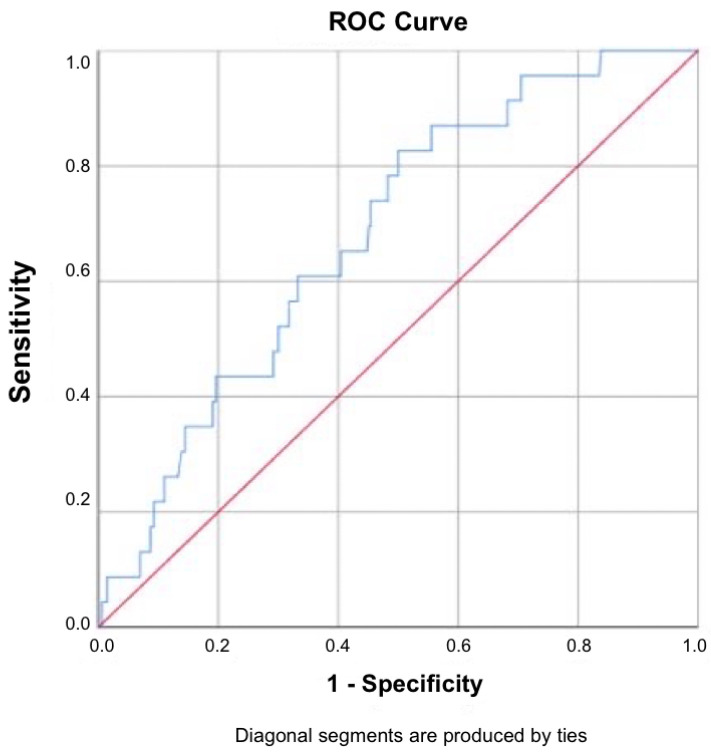
Receiver operating characteristic (ROC) curve for NLR in predicting cervical cancer. This ROC curve illustrates the diagnostic performance of the lymphocyte-to-monocyte ratio (LMR) in distinguishing patients with cervical cancer. Sensitivity (true-positive rate) is plotted on the *y*-axis, while 1—specificity (false-positive rate) is shown on the *x*-axis. The blue line represents the ROC curve derived from the study data, while the red diagonal line represents the reference line of no discrimination. The area under the curve (AUC) is 0.68007, indicating the moderate discriminative ability of LMR as a predictive biomarker for cervical malignancy.

The ROC curve demonstrated a moderate discriminatory ability, with an area under the curve (AUC) of 0.680. Using the Youden index, the optimal cutoff value was determined to be LMR < 4.49, yielding a sensitivity of 82.6% and a specificity of 50.0%. The Closest Top Left method proposed a slightly lower cutoff (LMR < 3.89), with a sensitivity of 60.9% and specificity of 66.8%. For the Youden-index cutoff (LMR = 4.49), sensitivity was 82.6%, specificity 50.0%, PPV 9.9%, and NPV 97.7%. For the Closest Top Left method (LMR = 3.89), sensitivity was 60.9%, specificity 66.8%, PPV 10.9%, and NPV 96.2%. The low PPV values reflect the low prevalence of histologically confirmed malignancy in the study cohort (~7.3%), whereas the high NPV values indicate the potential utility of LMR as a rule-out tool (Figure 7).

These results suggest that while LMR has limited value for confirming invasive disease due to low PPV, it may contribute as a rule-out tool in clinical decision-making, given its high NPV.

### 3.7. Statistical Analysis and Data Visualization

All statistical analyses were performed using IBM SPSS Statistics for Windows, Version 25.0 (IBM Corp., Armonk, NY, USA). Violin plots with median lines, overlaid with jittered individual data points, were generated using R (version 4.3.2) with the ggplot2 package. Missing data were handled via listwise deletion (complete-case analysis), and therefore, the number of valid cases differs between analyses, as reported in the corresponding tables and figures.

## 4. Discussion

Cervical cancer remains a major public health burden and is still too often diagnosed at advanced stages, when prognosis is markedly poorer [28]. Despite effective screening programs in high-income regions, socioeconomic and infrastructural barriers in many low- and middle-income countries (LMICs) limit early detection [29]. Consequently, there is an urgent need for simple, low-cost biomarkers that can stratify risk and guide further investigation [30]. 

Patients with cancer exhibited the lowest median LMR, and logistic regression analysis confirmed LMR as an inverse, independent predictor of malignancy (OR ≈ 0.62). Although the ROC curve showed only moderate discrimination (AUC = 0.68), the high negative predictive values at practical cutoffs (96–98%) indicate that a normal–high LMR can reliably exclude invasive disease, a property particularly useful where advanced diagnostics are scarce. Compared with other malignancies, the sensitivity (82.6%) and NPV (97.7%) in this study were higher than those reported for colon cancer (sensitivity 55.38%, specificity 88.77%, AUC 0.778, NPV 59.7%, and PPV 86.9%) [31]; osteosarcoma (sensitivity 59.9%, specificity 60.6%, AUC 0.603, NPV 59.2%, and PPV 61.3%) [32]; and breast cancer (sensitivity 57.35%, specificity 51.15%, AUC 0.515, NPV 69.0%, and PPV 38.8%) [33] and were comparable to non-small-cell lung cancer (sensitivity 81.72%, specificity 58.33%, AUC 0.745, NPV 45.2%, and PPV 88.4%) [34]. On the other hand, the specificity (50.0%) and PPV (9.9%) in this study were lower than in most of these malignancies, which may reflect differences in tumor biology, patient selection, and cutoff determination.

Post hoc analysis further demonstrated that this reduction in LMR was significant when comparing invasive carcinoma to low-grade lesions in cytology and to both low- and high-grade lesions in histology. This pattern likely reflects tumor-driven immune suppression, resulting in lower circulating lymphocytes, together with chronic inflammation leading to elevated monocytes, creating a systemic environment conducive to invasion and metastasis. The absence of notable LMR differences between LSIL and HSIL in this analysis suggests that systemic inflammatory markers, including LMR, are more relevant for identifying invasive cervical carcinoma than for distinguishing between preinvasive cytological grades. This reinforces the potential role of LMR as a rule-out tool for invasive disease rather than a discriminator among non-invasive lesions.

The findings in this study are consistent with studies on other malignancies linking low LMR to tumor progression and immune dysregulation. In colorectal cancer, a reduced pre-operative LMR correlated with a higher T-stage and worse overall survival [35]. Evidence from prostate, gastric, and multiple hematological cancers further supports LMR as a prognostic marker [14,36,37]. 

The utility of LMR in cervical cancer is less explored. Similar associations have been reported in cervical cancer cohorts treated surgically or with chemoradiotherapy, although data are from small cohorts or specific clinical contexts. Mu Xu et al. studied LMR combined with albumin in patients with IB–IIA cervical cancer and found that patients with low LMR had significantly worse relapse-free survival (RFS) and overall survival (OS) [38]. In another study, Hamilton Trinh studied patients with cervical cancer who underwent definitive radiochemotherapy and found an association between lower LMR values and worse overall survival and disease-free survival (DFS) [39]. Collectively, these studies and the data in this study suggest that LMR reflects the balance between antitumor immunity (lymphocyte-mediated cytotoxicity) and a tumor-promoting myeloid milieu driven by monocyte-derived tumor-associated macrophages. Diminished LMR, therefore, signals both immune suppression and an inflammatory microenvironment conducive to invasion and metastasis. A reduced lymphocyte-to-monocyte ratio (LMR) functions as an integrative biomarker capturing two fundamental pathological mechanisms in cancer progression. Firstly, it signifies immune suppression, as a decreased lymphocyte proportion reflects impaired adaptive immune surveillance and a weakened ability to generate cytotoxic responses against malignant cells. Secondly, it indicates the existence of a tumor-promoting inflammatory microenvironment, marked by increased monocyte counts capable of differentiating into tumor-associated macrophages (TAMs). These macrophages, together with various inflammatory mediators, establish a biochemical and structural environment that facilitates tumor cell invasion into adjacent tissues and supports metastasis through both hematogenous and lymphatic routes. In addition to its association with histological severity, LMR also varied according to HPV DNA status. In this cohort, patients with HPV DNA positivity had a lower median LMR (4.387) compared with HPV DNA-negative patients (5.377). This finding may reflect HPV-related immune modulation, as persistent high-risk HPV infection can induce chronic inflammation, leading to increased circulating monocyte counts and reduced lymphocyte-mediated immune surveillance, which together lower the LMR. Similar observations have been reported in advanced cervical cancer, where both LMR and HPV DNA status independently predicted survival outcomes [14]. Notably, in that study, the most favorable prognosis was seen in patients with both high LMR and HR-HPV positivity, and the poorest in those with low LMR and non-HR HPV. While this cross-sectional design does not permit causal inference, these results suggest that HPV-related immune responses may contribute to the lower LMR in HPV-positive individuals and that interpreting LMR alongside HPV DNA status could provide additional prognostic insight. These studies clearly show that inflammatory biomarkers reflect the patient’s immune status and tumor-associated inflammation; therefore, they can provide information about disease prognosis and potential reactions to treatments [14,37,38].

Compared with other blood-derived inflammatory markers, Vida et al. showed that NLR is significantly elevated in patients with malignant conization outcomes, with each unit increase in NLR raising cancer risk by 37.2% (OR = 1.372; *p* = 0.008) and an AUC of 0.734—slightly higher than the AUC of 0.680 observed for LMR in this study [7]. Similarly, Kalas et al. reported that elevated PLR values strongly correlated with histological severity, particularly in invasive carcinoma (Grade IV), yielding an AUC of 0.715 and a sensitivity/specificity of 65.2%/80.8% at a cutoff of 11.93. Despite varying cutoffs and mechanisms, all three markers demonstrated high negative but low positive predictive values, underscoring their shared utility in ruling out invasive disease. While NLR and PLR reflect systemic inflammation and thrombopoietic activation, LMR was specifically selected in the present study because it may more directly represent the immunologic balance between antitumor lymphocyte activity and tumor-promoting monocyte/macrophage activity, providing complementary insight into tumor–immune interactions [13].

From a prevention standpoint, the WHO cervical cancer elimination strategy encompasses primary (HPV vaccination), secondary (screening), and tertiary (treatment and survivorship) prevention [30]. LMR could enhance secondary and tertiary prevention in LMICs by triaging HPV-positive or cytology–minor-abnormality cases for colposcopy, flagging histology-borderline lesions that deserve closer surveillance, and informing prognostic discussions and follow-up intensity in confirmed cancer. Because LMR requires no specialized equipment and incurs a negligible extra cost, it is well suited to resource-constrained settings as an adjunctive marker. 

In summary, these findings suggest that the lymphocyte-to-monocyte ratio may serve as a practical, inexpensive complementary biomarker of disease severity in cervical lesions. LMR values were significantly lower in patients with more invasive carcinoma, and especially its negative predictive value makes it useful for potentially ruling out invasive disease. Even though its positive predictive value was low, the high negative predictive value means that a normal–high LMR can reliably exclude invasive carcinoma, reducing unnecessary invasive procedures in low-risk patients. These characteristics make it a valuable adjunct for ruling out invasion and guiding resource-efficient management, especially in regions where cervical cancer continues to claim the highest toll.

### 4.1. Strengths and Limitations

A major strength of this study is the integration of multiple data sources, including cytology, histology, and laboratory parameters, in a relatively large patient group. The use of standardized statistical methods, such as multivariable regression and ROC analysis, strengthens the validity of these findings. At the same time, there are some important limitations. The retrospective design limits the ability to prove cause-and-effect relationships; only associations can be observed. Additionally, there were missing values for some variables, for example, HPV status, which may have influenced the multivariable model. Lastly, since this was a single-center study, the results may not apply to all populations, and further research is needed to confirm them in a wider population with a larger sample size.

### 4.2. Implications for Practice

Due to its low cost, accessibility, and ease of calculation from routine blood tests, LMR holds promise as an adjunctive biomarker in the evaluation of cervical lesions. While not intended to replace cytology or histology, it may serve as a supportive tool for triaging patients, especially in settings with limited access to advanced diagnostics. In the future, LMR could aid clinical decision-making by identifying individuals who may benefit from closer follow-up or additional testing, thereby enhancing risk stratification and optimizing resource allocation in both high- and low-resource settings.

## 5. Conclusions

This study demonstrated that the lymphocyte-to-monocyte ratio (LMR) is significantly associated with both cytological and histological grades of cervical lesions. Lower LMR values were observed in patients with more severe lesions, indicating its potential role in risk stratification. The use of LMR, derived from routine complete blood count data, offers a simple and easily available tool. LMR may, therefore, be a practical, inexpensive complementary biomarker for predicting cervical lesion severity and excluding invasive carcinoma, supporting resource-efficient patient management.

## Figures and Tables

**Table 1:** Basic characteristics of the patient sample.

Characteristics (*n* = 374)	N (Range or %)
Total	374
Median age (years)	40 (23–78)
Median BMI	22.85 (14.64–46.48)
Median LMR	4.35 (0.32–18.52)
Cytology results	370
Grade I (negative)	10 (2.70%)
Grade II (LSIL, ASC-US)	73 (19.73%)
Grade III (HSIL, ASC-H, AGC, AIS)	270 (72.97%)
Grade IV (cancer)	17 (4.59%)
N/A	4
Conization results	369
Grade I (negative)	83 (22.49%)
Grade II (LSIL, ASC-US)	32 (8.67%)
Grade III (HSIL, ASC-H, AGC, AIS)	231 (62.60%)
Grade IV (cancer)	23 (6.23%)
N/A	5
HPV status	223
HPV positive	212 (95.07%)
HPV negative	11 (4.93%)

Basic characteristics. Categorical parameters are presented as n. Continuous data are presented as median (interquartile range).

**Table 2:** Number of patients (N) and median, mean, minimum, and maximum values of lymphocyte (%) and monocyte (%) counts and lymphocyte-to-monocyte ratio (LMR) according to cytology and conization results.

		Grade I (Negative)		Grade II (LSIL, ASC-US)		Grade III (HSIL, ASC-H, AGC, AIS)		Grade IV (Cancer)		Total	
	LMR	Cytol.	Coniz.	Cytol.	Coniz.	Cytol.	Coniz.	Cytol	Coniz.	Cytol.	Coniz.
	N	10	83	73	32	270	231	17	23	370	369
Lymphocyte (%)	Median	25.70	32.80	33.30	32.95	31.25	31.50	27.40	25.30	31.45	31.40
	Mean	28.14	32.06	33.11	34.02	31.76	31.83	27.95	25.10	31.76	31.65
	Minimum	14.30	1.52	8.20	21.10	2.00	2.00	13.40	7.30	2.00	1.52
	Maximum	47.50	47.50	53.70	53.00	60.60	60.60	39.30	39.30	60.60	60.60
Monocyte (%)	Median	7.20	7.10	7.50	7.25	7.00	7.00	7.70	7.10	7.00	7.00
	Mean	7.49	7.26	7.64	7.64	7.19	7.25	7.65	7.04	7.31	7.27
	Minimum	5.50	4.46	2.90	4.20	0.34	0.34	5.20	4.80	0.34	0.34
	Maximum	10.20	16.10	16.10	11.40	14.90	14.90	12.90	10.20	16.10	16.10
LMR (L/M)	Median	3.62	4.60	4.49	4.08	4.44	4.44	3.85	3.81	4.34	4.41
	Mean	3.75	4.61	4.76	4.62	4.64	4.70	3.79	3.69	4.60	4.61
	Minimum	1.83	0.32	1.04	2.54	0.80	0.62	1.74	0.80	0.80	0.32
	Maximum	5.94	8.70	18.52	7.06	13.28	18.52	5.75	6.18	18.52	18.52

**Table 3:** Pairwise comparisons of lymphocyte-to-monocyte ratio (LMR) across cytological and conization-based grades, using the Dunn–Bonferroni post hoc test following a significant Kruskal–Wallis result. Bonferroni-corrected *p*-values are shown for clarity.

Comparison Between Groups	Mann–Whitney U		Z-Score		Bonferroni Corrected *p*	
	Cytol.	Coniz.	Cytol.	Coniz.	Cytol.	Coniz.
Grade I vs. Grade II	1191.5	1220.5	−0.09	−0.082	1.00	1.00
Grade I vs. Grade III	8773.0	9205.0	−0.40	−0.261	1.00	1.00
Grade I vs. Grade IV	1189.0	561.0	−3.01	−2.770	0.02	0.03
Grade II vs. Grade III	3483.0	3663.0	−0.03	−0.063	1.00	1.00
Grade II vs. Grade IV	470.0	281.0	−2.33	−1.729	0.12	0.56
Grade III vs. Grade IV	1826.5	1856.5	−1.49	−2.495	0.82	0.047

**Table 4:** Logistic regression analysis evaluating lymphocyte-to-monocyte ratio (LMR) and age as independent predictors of cancer diagnosis.

Predictor	B	S.E.	Wald	df	Sig. (*p*)	Exp(B)	95% CI for Exp(B)
LMR	−0.48	0.18	7.36	1	0.01	0.62	[0.44–0.87]
Age	0.06	0.02	8.37	1	0.004	1.06	[1.02–1.10]
Constant	−3.29	1.13	8.43	1	0.004	0.04	[0.004–0.37]

## Data Availability

The data supporting the findings of this study can be obtained by contacting the corresponding author upon request.

## Abbreviations

The following abbreviations are used in this manuscript:

AGC	atypical glandular cell
AIS	adenocarcinoma in situ
ASC-H	atypical squamous cells, cannot exclude HSIL
ASC-US	atypical squamous cells, undetermined significance
AUC	area under the curve
BMI	body mass index
CI	confidence interval
CIN	cervical intraepithelial neoplasia
DFS	disease-free survival
HPV	human papillomavirus
HSIL	high-grade squamous intraepithelial lesion
LEEP	Loop Electrosurgical Excision Procedure
LMICs	low- and middle-income countries
LMR	lymphocyte-to-monocyte ratio
LSIL	low-grade squamous intraepithelial lesion
NLR	neutrophil-to-lymphocyte ratio
NPV	negative predictive value
OR	odds ratio
OS	overall survival
PLR	platelet-to-lymphocyte ratio
PPV	positive predictive value
RFS	recurrence-free survival
ROC	receiver operating characteristic
TAMs	tumor-associated macrophages
WHO	World Health Organization
